# The Microtubule-Associated Protein ASPM Regulates Spindle Assembly and Meiotic Progression in Mouse Oocytes

**DOI:** 10.1371/journal.pone.0049303

**Published:** 2012-11-13

**Authors:** Xiao-Ling Xu, Wei Ma, Yu-Bo Zhu, Chao Wang, Bing-Yuan Wang, Na An, Lei An, Yan Liu, Zhong-Hong Wu, Jian-Hui Tian

**Affiliations:** 1 Institute of Animal Husbandry and Veterinary Medicine, Beijing Municipal Academy of Agriculture and Forestry Sciences, Beijing, China; 2 Key Laboratory of Animal Genetics and Breeding of the Ministry of Agriculture, College of Animal Sciences and Technology, China Agricultural University, Beijing, China; 3 Department of Histology and Embryology, School of Basic Medical Sciences, Capital Medical University, Beijing, China; State Key Laboratory of Reproductive Biology, Institute of Zoology, Chinese Academy of Sciences, China

## Abstract

The microtubule-associated protein ASPM (abnormal spindle-like microcephaly-associated) plays an important role in spindle organization and cell division in mitosis and meiosis in lower animals, but its function in mouse oocyte meiosis has not been investigated. In this study, we characterized the localization and expression dynamics of ASPM during mouse oocyte meiotic maturation and analyzed the effects of the downregulation of ASPM expression on meiotic spindle assembly and meiotic progression. Immunofluorescence analysis showed that ASPM localized to the entire spindle at metaphase I (MI) and metaphase II (MII), colocalizing with the spindle microtubule protein acetylated tubulin (Ac-tubulin). In taxol-treated oocytes, ASPM colocalized with Ac-tubulin on the excessively polymerized microtubule fibers of enlarged spindles and the numerous asters in the cytoplasm. Nocodazole treatment induced the gradual disassembly of microtubule fibers, during which ASPM remained colocalized with the dynamic Ac-tubulin. The downregulation of ASPM expression by a gene-specific morpholino resulted in an abnormal meiotic spindle and inhibited meiotic progression; most of the treated oocytes were blocked in the MI stage with elongated meiotic spindles. Furthermore, coimmunoprecipitation combined with mass spectrometry and western blot analysis revealed that ASPM interacted with calmodulin in MI oocytes and that these proteins colocalized at the spindle. Our results provide strong evidence that ASPM plays a critical role in meiotic spindle assembly and meiotic progression in mouse oocytes.

## Introduction

In mammals, meiosis is unique to germ cells and is critical for sexual reproduction [1]. In females, meiosis occurs in cells known as oogonia. Each oogonium that initiates meiosis divides twice to form a single oocyte. Oocytes from all mammalian species are blocked in the ovary in prophase of meiosis I until meiosis resumes. During arrest, they contain a large centrally located nucleus called the germinal vesicle (GV). After individual sexual maturation, the oocyte is triggered to resume meiosis by gonadotropin stimulation or other factors. Then, the GV undergoes breakdown (GVBD), chromatin is condensed into chromosomes, and the typical barrel-shaped spindle begins to form around the chromosomes. Spindle formation is followed by two consecutive asymmetric divisions, resulting in the formation of a large haploid oocyte and small polar bodies [2].

These asymmetric cell divisions ensure the maximal retention of the maternal cytoplasmic components that are required for early development [3]. Asymmetric cell division is closely related to cell polarity, spindle position and spindle orientation. The polarity of the mouse oocyte affects the migration of the spindle to the cell cortex and the polarization of the cortex, the former of which relies on microfilaments (i.e., actin fibers). Microfilaments are polymers formed by globular actin monomers. So far, three main types of microfilament nucleation factors have been found: the Actin-related protein 2/3 complex and the Spire and Formin proteins. Meanwhile, studies of Mos, a member of the small G protein superfamily, have shown that this protein plays important roles in spindle movement. In the mouse, the cortex of GV-stage oocytes has no apparent polarity but becomes polarized during maturation [4]. The molecular details of oocyte cortical polarization are only beginning to emerge.

Meanwhile, meiotic spindle assembly and migration is crucial for meiotic progression and the asymmetry of the meiotic division. The spindle, which is mainly composed of microtubules, is an essential cellular structure that is responsible for the accurate segregation of chromosomes in both mitosis and meiosis [5]. Unlike the mitotic spindles of somatic cells, which have astral microtubules and centrosomes and are diamond-shaped, the spindles of meiotic oocytes are barrel-shaped and have microtubule organizing centers (MTOCs) that functionally replace centrosomes and form de novo from a cytoplasmic microtubule network during prophase [6]. MTOCs are essential for meiotic spindle assembly. Recently, it has been reported that PKCδ [7], BRCA1 [5], LGN [8], and Nedd1 [9] play critical roles in meiotic spindle organization and spindle stability. However, many of the molecules involved in the complex process of meiotic spindle assembly and positioning during meiosis remain to be identified.

ASPM is a structurally and functionally conserved microtubule associated protein. In *Drosophila*, ASPM is enriched at the poles of meiotic and mitotic spindles and localizes to the minus ends of central spindle microtubules, and it is involved in microtubule organization during spindle formation and cytokinesis [10]. In *C. elegans*, ASPM and calmodulin work together to promote meiotic spindle organization and the accumulation of LIN-5 at meiotic and mitotic spindle poles. These proteins also contribute to meiotic spindle positioning in conjunction with the microtubule motor dynein [11]. CDK-1 blocks rotation by inhibiting dynein association with microtubules and with LIN-5-ASPM-1 at meiotic spindle poles, while the Anaphase-promoting complex (APC) promotes spindle rotation by inhibiting CDK-1 [12]. In mammals, ASPM is involved in the regulation of neurogenesis, and mutations in ASPM have been linked to primary autosomal recessive microcephaly (MCPH) [13], [14]. The predicted ASPM protein has two distinct regions: N-terminal putative calponin-homology (CH) domains and a large block of IQ motifs, which are commonly involved in actin binding and mediating interactions with calmodulin and calmodulin-related proteins, respectively [15], [16]. ASPM is widely expressed in fetal and adult tissues and is upregulated in malignant cells [17]. In human U2OS osteosarcoma cells, ASPM knockdown alters the positioning of the mitotic spindle from parallel to perpendicular to the substrate, effectively altering the division symmetry from symmetrical to asymmetrical, thus inducing cytokinesis failure and apoptosis [18]. Meanwhile, in human U2OS cells, the downregulation of endogenous ASPM by siRNA decreases the levels of endogenous BRCA1 protein, which indicates that ASPM may be involved in mitotic spindle function, possibly through the regulation of BRCA1 [19]. Recently, it has been reported that BRCA1 plays an important role in meiotic spindle organization in mouse oocytes [5].

In ASPM mutant mice, truncated ASPM proteins similar to those that cause microcephaly in humans fail to localize to the midbody during M phase. This causes not only mild microcephaly but also a massive loss of germ cells, resulting in a severe reduction in testis and ovary size accompanied by low fertility [20]. This observation led us to further study the function of ASPM in mouse oocyte meiosis. First, we examined the subcellular localization of ASPM during oocyte maturation. We then analyzed the behavior of ASPM during spindle-perturbing drug treatment and evaluated the effects of gene-specific morpholino-mediated ASPM knockdown on meiotic spindle assembly and meiotic progression. Furthermore, we identified ASPM-interacting proteins in MI oocytes by coimmunoprecipitation combined with mass spectrometry and western blot analysis. The results provide strong evidence showing that ASPM plays a critical role in meiotic spindle assembly and meiotic progression.

## Results

### Expression and Subcellular Localization of ASPM During Mouse Oocyte Meiotic Maturation

**Figure 1:**
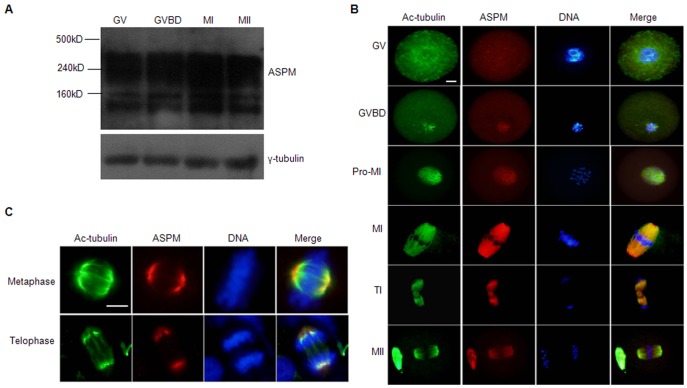
Expression and subcellular localization of ASPM in mouse oocytes and MEFs. (**A**) Total proteins from 100 GV, GVBD, MI and MII oocytes were separated and blotted with antibody against mouse ASPM. (**B**) GV, GVBD, pro-MI, TI, and MII oocytes costained with Ac-tubulin (green), ASPM (red) and DAPI (blue). Bar = 10 µm. (**C**) Metaphase and telophase MEFs co-labeled with Ac-tubulin (green), ASPM (red) and DAPI (blue). Bar = 10 µm.

To investigate the role of ASPM during mouse oocyte meiotic maturation, we examined its expression and subcellular localization. An analysis of ASPM expression in mouse oocytes during meiosis by western blot revealed four bands, corresponding to 364, 212, 130, and 70 kDa,each with similar expression levels among different stages (GV, GVBD, MI, MII) (Figure 1A). Therefore, ASPM was expressed in oocytes from the GV to MII stages.

To examine the subcellular localization of ASPM, we performed immunofluorescence staining on oocytes at different stages of maturation and also on mouse embryonic fibroblasts (MEFs). At the GV stage, ASPM was distributed evenly in the cytoplasm. At GVBD, when chromatin was condensing and microtubules were beginning to polymerize, ASPM congregated around the chromatin and colocalized completely with the microtubules. When oocytes progressed to pro-MI and MI, ASPM was concentrated at the total spindle apparatus; for MII-arrested oocytes, ASPM was concentrated at the spindle and the polar body (Figure 1B). Throughout meiosis, Ac-tubulin co-immunostained with ASPM, suggesting that ASPM might play a role in spindle organization. However, in metaphase and telophase somatic MEFs, ASPM localized to the spindle poles (Figure 1C), distinct from its localization in the oocytes.

### Localization of ASPM in Mouse Oocytes Treated with Spindle-Perturbing Agents

**Figure 2:**
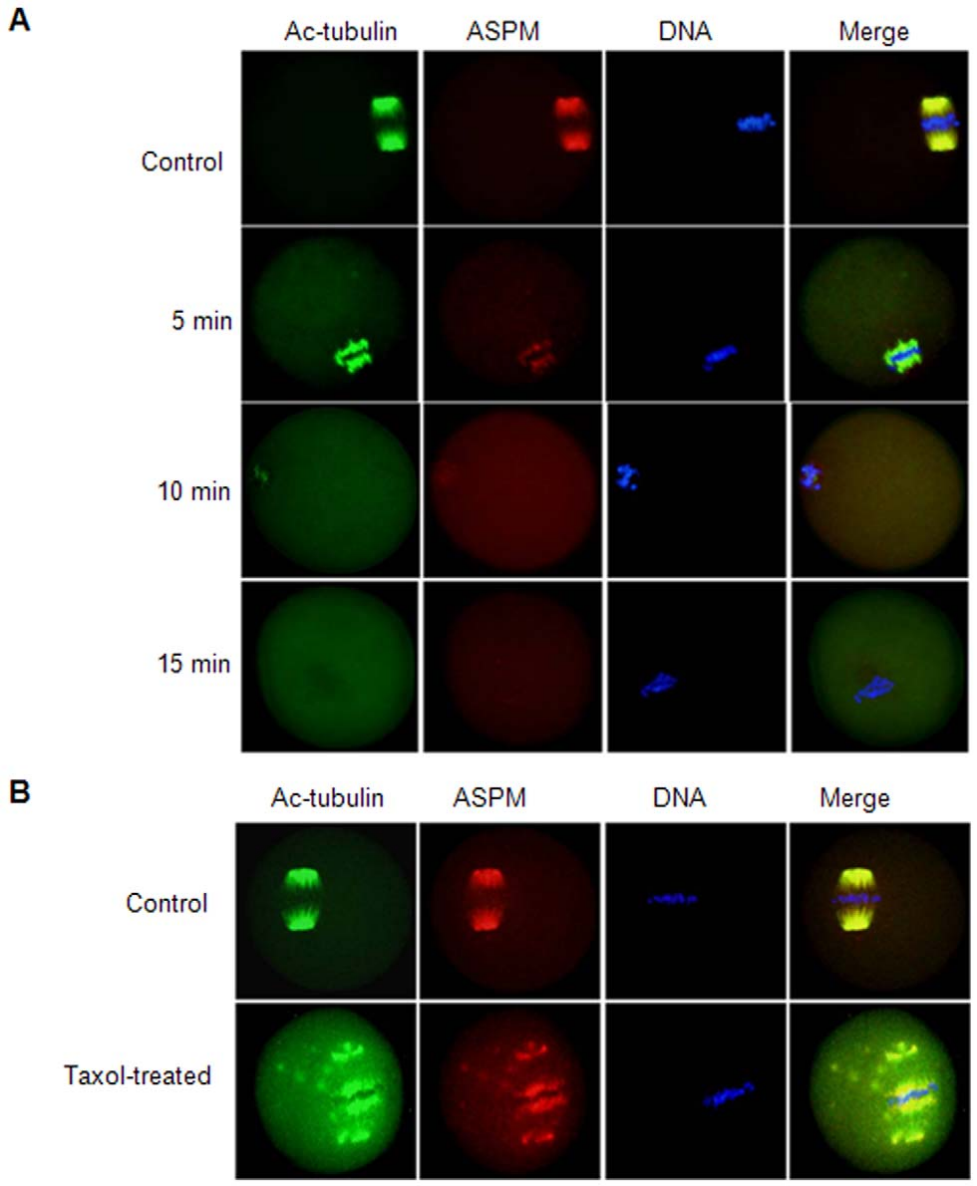
Localization of ASPM in mouse oocytes treated with spindle-perturbing agents. (**A**) MI oocytes were incubated with 20 µg/ml nocodazole for 5, 10 or 15 min and then costained with Ac-tubulin (green), ASPM (red) and DAPI (blue). Bar = 10 µm. (**B**) MI oocytes were incubated with 10 µM taxol for 45 min and then costained with Ac-tubulin (green), ASPM (red) and DAPI (blue). Bar = 10 µm.

To clarify the correlation between ASPM and microtubules, we treated MI oocytes with spindle-perturbing drugs (nocodazole and taxol). First, we treated the MI oocytes for 5, 10 and 15 min with the microtubule-depolymerizing agent nocodazole. We found that the microtubules were gradually disassembled with an increase in treatment time, and no intact spindles were observed in the oocytes after 15 min; ASPM remained colocalized with Ac-tubulin during the entire process (Figure 2A).

Next, the MI oocytes were treated with the microtubule-stabilizing reagent taxol (10 µM) for 45 min. After treatment, the microtubule fibers in taxol-treated oocytes were excessively polymerized, and numerous asters were observed in the cytoplasm. Again, ASPM remained colocalized with Ac-tubulin (Figure 2B).

### Downregulation of ASPM by a Gene-specific Morpholino Disrupts Meiotic Spindle Assembly and Meiosis Procession

**Figure 3:**
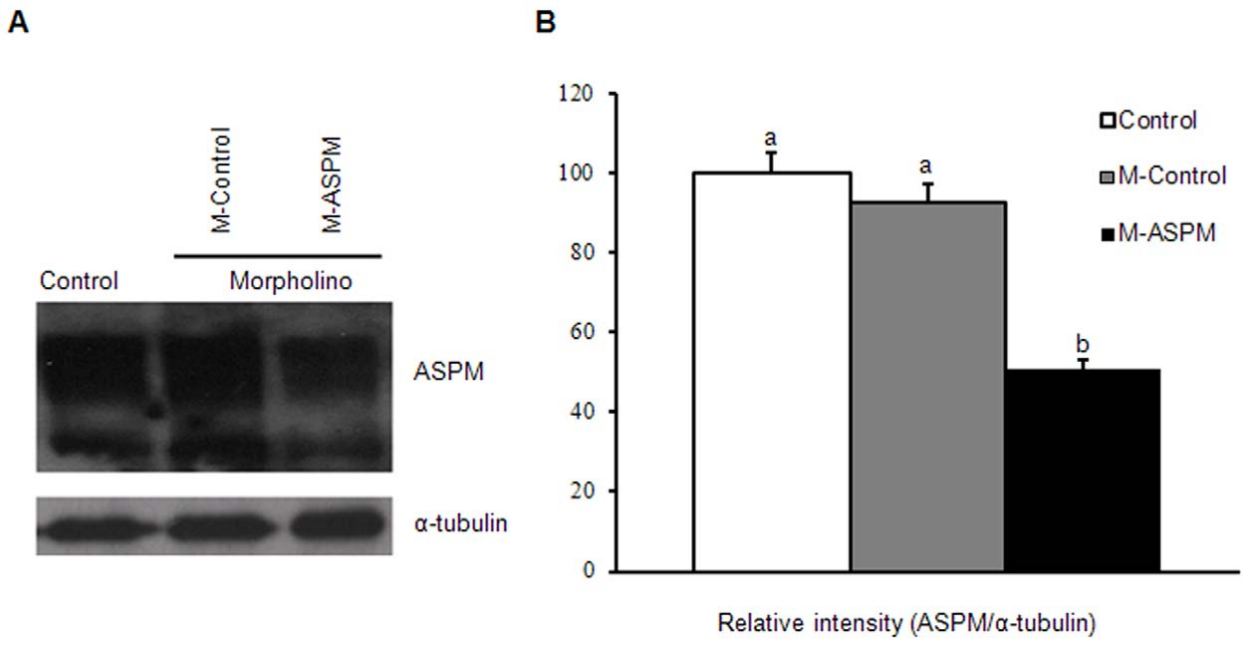
ASPM expression was successfully reduced in oocytes by ASPM morpholinos. (**A**) Western blot of ASPM analysis revealed a 49.14% decrease in the ASPM expression level following ASPM morpholino injection relative to the uninjected control and control morpholino groups. The same blot revealed comparable levels of α-tubulin in all 3 groups. Each sample contains 100 oocytes. (**B**) Relative intensity of the bands of ASPM or α-tubulin was analyzed with Image J software.

Gene-specific morpholino oligonucleotides have proven to be an effective approach for gene knockdown in mouse oocytes [21], [22]. Western blot analyses revealed that ASPM protein expression was reduced by 49.14% in mouse oocytes by microinjecting ASPM-specific morpholino oligonucleotides (Figure 3).

After 18 h of culture, DAPI-labeled DNA configurations were assessed to determine the progression of meiosis in each group of mouse oocytes (Table 1). Of the total oocytes evaluated, 83.48% and 70.85% progressed to MII in the uninjected control group and the control morpholino group, respectively; however, in the ASPM morpholino group, only 19.51% of the oocytes progressed to MII, while most remained at MI. This result indicated that the decrease in the expression of ASPM greatly disrupted the meiotic progression.

**Table 1:** Meiotic progression.

Group	Total Oocytes	MII% (n)
Control	266	83.48±3.87%^a^ (220)
M-Control	203	70.85±6.40%^a^ (145)
M-ASPM	222	19.51±6.61%^b^ (52)

Note: The progression of meiosis was evaluated at the end of 18-hour culture. The number of oocytes at the MII stage was determined by DAPI-labeled DNA configuration. The values represent the mean values ± SEM, with oocyte numbers in brackets, of 3 independent replicates with a minimum of 30 oocytes in each group. Different letters denote significant differences (P<0.05) between groups.

**Figure 4:**
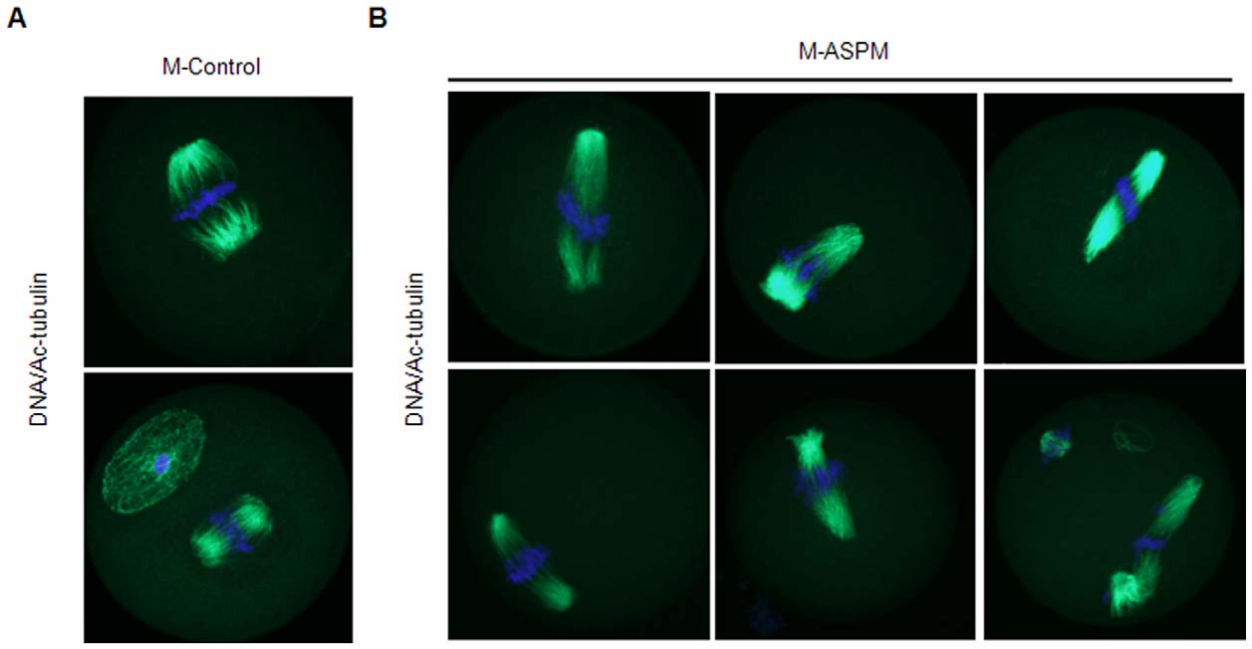
Downregulation of ASPM expression by morpholinos disrupts meiotic spindle organization. (**A**) and (**B**) Oocytes injected with morpholino control or ASPM morpholinos were matured for 18 h and stained with DAPI (blue) and Ac-tubulin (green). Bar = 10 µm.

To further evaluate the functional effects of the downregulation of ASPM expression, the oocytes were examined by immunofluorescence. As shown in Figure 4, ASPM depletion led to abnormal spindle assembly. Elongated spindles were frequently observed in oocytes with reduced expression of ASPM, while disorganized spindles lacking intact poles were also found (Figure 4B). Abnormal meiotic MI and MII spindle organization was observed in 13.33% and 11.06% of the uninjected control oocytes and in 4.34% and 12.45% of the oocytes injected with morpholino control, respectively. In contrast, in ASPM morpholino-injected oocytes, 45.28% and 47.80% of oocytes exhibited abnormal meiotic MI and MII spindle assembly, respectively (Table 2). There were significant differences between the morpholino control and the ASPM morpholino-injected group (P<0.05). These results indicate that ASPM is required for meiotic spindle assembly during both meiotic divisions.

**Table 2:** Disrupted meiotic spindle configurations.

Group	Total Oocytes	MI Abnormal Rate (n)	MII Abnormal Rate (n)
Control	103	13.33±11.54%^A^ (18)	11.06±4.15%^a^ (77)
M-Control	128	4.34±3.81%^A^ (34)	12.45±2.23%^a^ (83)
M-ASPM	177	45.28±10.59%^B^ (56)	47.80±12.71%^b^ (33)

Note: Meiotic spindle configurations were evaluated by immunofluorescence analysis of individual oocytes that were fixed at the end of an 18-hour culture. Spindle microtubules were detected with anti-Ac-tubulin, and the DNA was labeled with DAPI. The values represent the mean values ± SEM, with oocyte numbers in brackets, of 3 independent replicates. Different letters denote significant differences (P<0.05) between group.

**Figure 5:**
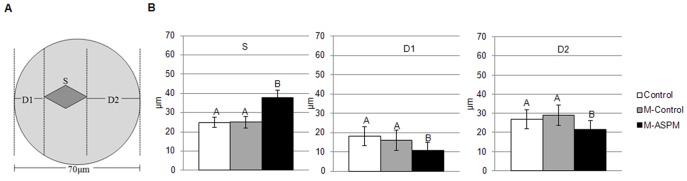
Downregulation of ASPM expression by morpholinos perturbs the asymmetrical division of mouse oocytes. (**A**) A cartoon shows an oocyte in the metaphase of meiosis I. (**B**) S, spindle length. D1, the distance between the closer spindle pole and the cortex. D2, the distance between the further spindle pole and the cortex. In the histogram, the different colors representing the different groups are listed at the right. Different letters denote significant differences (P<0.05) between groups.

To quantitatively compare the behavior of the spindle in control oocytes and in ASPM morpholino-injected oocytes, we measured the spindle length (S), the distance from the spindle pole to the closer cortex (D1), and the distance from the other spindle pole to the further cortex (D2) at the end of culture, as previously described [23] (Figure 5A). Both the control and morpholino-injected control oocytes had similar S values in MI-stage oocytes (Control = 24.91±2.70 µm, M-control = 24.99±2.99 µm, average ± STDEV). However, in the oocytes in the ASPM morpholino-injected group, S was significantly larger (M-ASPM = 37.75±3.80 µm, average±STDEV) (Figure 5B). Similarly, the uninjected and injected control oocytes had similar values of D1 (Control = 18.24±4.90 µm, M-Control = 15.97±5.21 µm, average±STDEV) and D2 (Control = 26.87±5.06 µm, M-Control = 29.04±5.27 µm, average±STDEV), both of which were significantly different from the ASPM morpholino-injected group (D1, M-ASPM = 10.78±4.31 µm and D2, M-ASPM = 21.48±4.69 µm, average ± STDEV).

### The Spindle Protein Calmodulin Co-immunoprecipitated with ASPM

**Figure 6:**
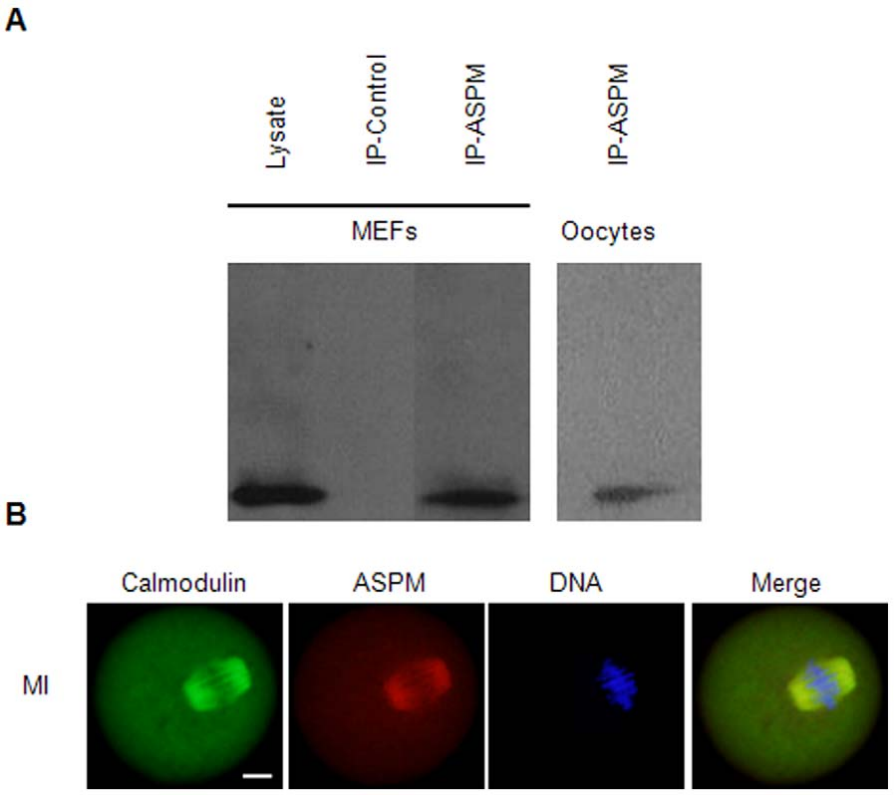
ASPM co-immunoprecipitated with calmodulin and colocalized with calmodulin on MI-stage spindles. (**A**) Co-immunoprecipitation studies with MEFs and mouse MI-stage oocytes identified one band corresponding to calmodulin (20 kDa, arrowhead) in the ASPM immunoprecipitate. (**B**) MI oocytes costained with calmodulin (green), ASPM (red) and DAPI (blue). Bar = 10 µm.

The detection of ASPM at the meiotic spindle and downregulation of ASPM led to an abnormal meiotic spindle and inhibited meiotic progression prompted us to investigate whether ASPM co-localized with specific spindle-associated proteins to control spindle assembly. Lysates from MEFs and mouse MI-stage oocytes were used for the studies. The immunoprecipitation (IP) of ASPM from lysates followed by mass spectrometry identified a peptide absent from control IPs that corresponded to calmodulin (data not shown). Further western blot analysis confirmed the expression of calmodulin as a 20-kDa band in the IP elution lane; the same bands were detected in control MEF cell lysates but not in the IP-control lane (Figure 6A).

These results indicated that ASPM co-immunoprecipitated with calmodulin in the MEFs and in the mouse oocytes. Therefore, we next tested the localization of ASPM and calmodulin during mouse oocyte meiosis. We found that ASPM and calmodulin colocalized at the spindles of MI and MII oocytes (Figure 6B).

## Discussion

In this study, we provide the first evidence that ASPM is a conserved microtubule-associated protein that plays an essential role in the control of spindle organization during mouse oocyte meiotic maturation. The perturbation of ASPM function causes meiotic spindle assembly defects, and first polar body extrusion (PBE) greatly decreased when ASPM was partially inhibited.

Previous reports have shown that ASPM colocalizes with γ-tubulin at the spindle poles during mitosis in *Drosophila* [24] and human U2OS, HeLa and HaCaT cells [19]. Therefore, we examined the localization of ASPM in mouse oocytes at different stages by immunofluorescence. Our results showed that ASPM and the spindle microtubule protein Ac-tubulin were colocalized to the entire spindle at MI and MII and also overlapped at the midbody in telophase I (Figure 1B). This localization pattern may indicate that ASPM regulates spindle assembly during mouse oocyte maturation. However, this was inconsistent with the previously published observations of ASPM localization during mitosis, so we investigated the localization in mouse MEFs to ensure the reliability of the ASPM antibody. In MEFs, ASPM localized to the spindle poles during mitotic metaphase (Figure 1C). Therefore, we deduced that ASPM played different roles in mitosis and meiosis. Meanwhile, this was the first demonstration of ASPM localization in mouse oocytes.

The subcellular colocalization of Ac-tubulin and ASPM during meiotic maturation prompted us to further explore the relationship between microtubules and ASPM using the spindle-perturbing drugs nocodazole and taxol. After nocodazole treatment, microtubules were depolymerized; ASPM and Ac-tubulin disappeared from the spindle upon complete spindle collapse. After taxol treatment, microtubule fibers were excessively polymerized and formed a large spindle, together with numerous asters in the cytoplasm of treated oocytes, ASPM and Ac-tubulin colocalized to the spindle and cytoplasmic asters. Therefore, the localization pattern of ASPM is similar to that of other proteins that are involved in spindle formation, such as septin1, which is most likely attached to the spindle components, perhaps participating in meiotic spindle assembly and maintenance [25].

To dissect the role of ASPM in mouse oocyte maturation, we used gene-specific morpholino injection to knock down ASPM protein expression and assess the loss of function phenotype. Western blot confirmed that the ASPM protein level was reduced by 49.14% after ASPM morpholino injection. Previous reports showed that some conserved proteins involved in the regulation of spindle organization and spindle assembly are relatively stable, such as LGN and p38α, but even moderate downregulation can cause functional effects [8], [22]. After morpholino injection, a large proportion of ASPM-ablated oocytes exhibited spindle assembly defects. The predominant phenotypes included spindle elongation and disorganized spindle poles. In *C. elegans*, ASPM, as a novel LIN-5 binding partner, acts together with calmodulin to promote meiotic spindle organization [11]. The RNAi knockdown of ASPM and calmodulin resulted in a specific loss of LIN-5 from meiotic spindles and caused obvious defects in MI and MII. Meanwhile, ASPM was no longer detected at meiotic and mitotic spindles after RNAi of calmodulin, so calmodulin is needed for the localization of ASPM [11]. LIN-5 (an ortholog of mammalian NuMA), a microtubule crosslinker, and dynein/dynactin, a multi-subunit microtubule minus-end-directed motor complex, regulate spindle length [26]. Our data showed that during MI, ASPM co-immunoprecipitated with calmodulin and that these two proteins colocalized to the spindle. It has been demonstrated that calmodulin binding to IQ motifs induces a conformational change that regulates the binding of actin to the amino-terminal CH domains [27]. CH and IQ domains were first discovered in motor proteins such as unconventional myosins [28]. A recent study of myosin X function in early *Xenopus* embryo mitosis indicated that unconventional myosin ensured pole integrity and normal spindle length by localizing to poles and exerting pulling forces on actin filaments within the spindle and also reported increased spindle length upon knockdown of myosin X, presumably due to the activation of the spindle assembly checkpoint (SAC) [29].

In addition, the downregulation of ASPM significantly decreased the PBE rate; most of the oocytes remained blocked in MI. In *C. elegans*, the same phenomenon was observed in ASPM-deleted oocytes [11]. In *Drosophila,* abnormal spindle (asp) contains six consensus sites for phosphorylation by p34cdc2 and four consensus sites for phosphorylation by MAP kinase [24], and *Drosophila* asp mutants exhibit mitotic metaphase checkpoint arrest with abnormal spindle poles [30]. Based on these data, we hypothesize that most of the oocytes blocked in MI after the downregulation of ASPM may be attributed to the metaphase checkpoint arrest and spindle assembly checkpoint (SAC) malfunction, blocking the activation of the anaphase-promoting complex. However, further studies will be needed to dissect the molecular machinery and determine how the perturbation of ASPM affects SAC function.

Taken together, our ASPM subcellular localization, interaction and function data strongly suggest that ASPM plays a critical role in meiotic spindle organization and meiotic progression. Understanding the nature and function of ASPM on mouse oocyte meiosis will enrich our knowledge of the regulatory mechanisms involved in spindle organization and lay the foundation for further exploration of the intricacies of the meiotic cell cycle.

## Materials and Methods

### Ethics Statement

The experimental procedure was approved by the Animal Care Commission of the College of Animal Sciences and Technology, China Agricultural University. The adult (8-week-old) C57BL/6J female and DBA/2J male mice purchased from laboratory animal research center of the Academy of Military Medical Sciences. They were housed in a standard animal facility under conditions of controlled temperature (20°C) and photoperiod (a 12∶12-hour light/dark schedule), with food and water provided ad libitum.

### Chemicals

All chemicals were purchased from Sigma-Aldrich (Sigma, USA) unless otherwise indicated.

### Oocyte Collection and Culture

Oocytes were isolated from 21- to 23-day old female B6D2F1 (C57BL/6J×DBA/2J F1) mice that had been injected with 5IU of pregnant mare serum gonadotropin (PMSG) to stimulate pre-ovulatory follicle development. The oocytes were collected in M2 medium with or without 2.5 mM milrinone to keep the oocytes at the GV stage. The oocytes were then washed thoroughly and cultured in M2 under liquid paraffin oil at 37°C in an atmosphere containing 5% CO_2_ in air. At different time points during the culture, the oocytes were collected for immunofluorescence, drug treatment, microinjection and western blot.

### Nocodazole and Taxol Treatment of MI Oocytes

MI-stage oocytes were treated with nocodazole or taxol. For nocodazole treatment, 10 mg/ml nocodazole in dimethylsulfoxide (DMSO) stock was diluted in M2 medium to a final concentration of 20 µg/ml, and oocytes were incubated in this medium for 5, 10 or 15 min; for taxol treatment, 5 mM taxol in DMSO stock was diluted in M2 medium to a final concentration of 10 µM, and MI-stage oocytes were incubated in this medium for 45 min at 37°C in an atmosphere of 5% CO_2_ in air. After treatment, the oocytes were washed thoroughly and prepared for immunofluorescence. Control oocytes were treated with the same concentration of DMSO in the medium before examination.

### Immunofluorescence and Confocal Microscopy

For the immunofluorescence staining of ASPM, calmodulin and Ac-tubulin, oocytes were fixed in 4% paraformaldehyde in PBS (pH 7.4) and permeabilized with 0.5% Triton X-100 for 45 min at 37°C. After three washes with 0.1% PVA/PBS at 37°C for 5 min, the oocytes were blocked in PBS supplemented with 1% BSA for 1 h and incubated overnight at 4°C with 1∶500 rabbit anti-ASPM antibody (purchased from the Bethyl Laboratories, USA), 1∶200 mouse anti-calmodulin antibody (purchased from the Invitrogen, USA) or 1∶10000 anti-Ac-tubulin antibody. The oocytes were then washed three times in 0.1% PVA/PBS containing 0.02% Triton X-100 at 37°C for 20 min each and labeled with Alexa Fluor 594 Goat Anti-Rabbit or Alexa Fluor 488 Goat Anti-Mouse (Molecular Probes) for 1 h at room temperature in the dark. After another three washes in 0.1% PVA/PBS containing 0.02% Triton X-100 at 37°C for 20 min each, the oocytes were costained with DAPI (Sigma). Finally, the oocytes were mounted on glass slides and examined with a confocal laser scanning microscope (Zeiss LSM 510 META, Germany).

### Co-immunoprecipitation

Co-immunoprecipitation studies were undertaken to determine whether endogenous ASPM interacts with specific proteins. Lysates were prepared from MEFs and mouse MI-stage oocytes. Analysis was performed using the ProFound™ Mammalian Co-Immunoprecipitation Kit (Pierce, Rockford, IL) in accordance with the manufacturer’s instructions. Briefly, rabbit anti-ASPM antibody was immobilized on the coupling gel. Non-related rabbit IgG (Sigma) was used as an immunoprecipitation control. The co-immunoprecipitation complex was eluted and processed for mass spectrometry and western blot analysis.

### Western Blot

Denuded GV-, GVBD-, MI- and MII-stage oocytes were collected and frozen in 2X Laemmli buffer (Bio-Rad) with protease inhibitors. Prior to analysis, the samples were thawed and subsequently heated to 100°C for 5 min. The proteins were separated on 7.5% or 12% acrylamide gels containing 0.1% SDS and then transferred onto hydrophobic PVDF membranes (Amersham, Piscataway, NJ). The membranes were blocked with 5% non-fat dried milk in TBS containing 0.05% (v/v) Tween-20 overnight at 4°C and incubated with a diluted rabbit antibody against ASPM (1∶2000) and mouse antibody against calmodulin (1∶1000) for 2 h at room temperature, followed by three (20-minute)washes in TBS containing 0.05% (v/v) Tween-20. A peroxidase-conjugated secondary antibody (Jackson ImmunoResearch, West Grove, PA) was added for 1 h, and protein bands were then detected using an ECL-plus system (Amersham, Piscataway, NJ). The densitometries of bands were analyzed with Image J software.

### Knockdown of ASPM Expression in Oocytes by Gene-specific Morpholinos

To assess ASPM function in oocyte meiosis, ASPM-specific morpholinos (TAGAAGCCGAGCCACCAGAGGTCAT, Gene Tool), 25 nucleotides in length, were used to knockdown ASPM translation levels in oocytes. Control groups included: (i) non-injected oocytes that were subject to the same culture conditions and (ii) oocytes injected with standard control morpholinos (CCTCTTACCTCAGTTACAATTTATA, Gene Tool). For each group, 10pl of 1 mM morpholino solution (Gene Tool) was microinjected directly into the cytoplasm of denuded, fully grown oocytes arrested at GV in medium supplemented with 2.5 µg/ml milrinone. Sterile Femtotip capillaries and a FemtoJet microinjector (Eppendorf, Westbury, NY) were used to standardize the injection volumes. Following oocyte microinjection, oocytes were maintained in GV arrest for 30 h. The oocytes were subsequently washed thoroughly, transferred to fresh medium and cultured for an additional 18 h. Finally, the oocytes were collected for further western blot and immunostaining analysis.

### Statistical Analysis

All data are presented as the mean percentages (± SEM) of a minimum of 3 independent experimental replicates. The different groups were analyzed by one-way ANOVA using the program SAS (SAS Institute, Cary, NC, USA). Significance was assigned at P<0.05.

## References

[1] Shin YH, Choi Y, Erdin SU, Yatsenko SA, Kloc M, et al (2010) Hormad1 mutation disrupts synaptonemal complex formation, recombination, and chromosome segregation in mammalian meiosis.PLoS Genet. 6: e1001190. 10.1371/journal.pgen.1001190 PMC2973818 21079677

[2] Azoury J, Lee KW, Georget V, Rassinier P, Leader B, et al (2008) Spindle positioning in mouse oocytes relies on a dynamic meshwork of actin filaments. Curr Biol 18: 1514–9. 18848445 10.1016/j.cub.2008.08.044

[3] Bezanilla M, Wadsworth P (2009) Spindle positioning: actin mediates pushing and pulling. Curr Biol 19: R168–9. 19243693 10.1016/j.cub.2008.12.026 PMC2848404

[4] Maro B, Verlhac MH (2002) Polar body formation: new rules for asymmetric divisions. Nat Cell Biol 4: E281–3. 12461532 10.1038/ncb1202-e281

[5] Xiong B, Li S, Ai JS, Yin S, Ouyang YC, et al (2008) BRCA1 is required for meiotic spindle assembly and spindle assembly checkpoint activation in mouse oocytes. Biol Reprod 79: 718–26. 18596218 10.1095/biolreprod.108.069641

[6] Schuh M, Ellenberg J (2007) Self-organization of MTOCs replaces centrosome function during acentrosomal spindle assembly in live mouse oocytes. Cell130: 484–98. 10.1016/j.cell.2007.06.025 17693257

[7] Ma W, Baumann C, Viveiros MM (2010) NEDD1 is crucial for meiotic spindle stability and accurate chromosome segregation in mammalian oocytes.DevBiol. 339: 439–50. 10.1016/j.ydbio.2010.01.009 20079731

[8] Guo X, Gao S (2009) Pins homolog LGN regulates meiotic spindle organization in mouse oocytes. Cell Res19: 838–48. 10.1038/cr.2009.54 19434098

[9] Ma W, Koch JA, Viveiros MM (2008) Protein kinase C delta (PKCdelta) interacts with microtubule organizing center (MTOC)-associated proteins and participates in meiotic spindle organization.Dev. Biol320: 414–25. 10.1016/j.ydbio.2008.05.550 18602096

[10] Wakefield JG, Bonaccorsi S, Gatti M (2001) The drosophila protein asp is involved in microtubule organization during spindle formation and cytokinesis. J Cell Biol153: 637–48. 10.1083/jcb.153.4.637 PMC2192390 11352927

[11] van der Voet M, Berends CW, Perreault A, Nguyen-Ngoc T, Gönczy P, et al (2009) NuMA-related LIN-5, ASPM-1, calmodulin and dynein promote meiotic spindle rotation independently of cortical LIN-5/GPR/Galpha. Nat Cell Biol 11: 269–77. 19219036 10.1038/ncb1834

[12] Ellefson ML, McNally FJ (2011) CDK-1 inhibits meiotic spindle shortening and dynein-dependent spindle rotation in C. elegans. J Cell Biol193: 1229–44. 10.1083/jcb.201104008 PMC3216336 21690306

[13] Roberts E, Hampshire DJ, Pattison L, Springell K, Jafri H, et al (2002) Autosomal recessive primary microcephaly: an analysis of locus heterogeneity and phenotypic variation. J Med Genet39: 718–721. 10.1136/jmg.39.10.718 PMC1734986 12362027

[14] Bond J, Roberts E, Mochida GH, Hampshire DJ, Scott S, et al (2002) ASPM is a major determinant of cerebral cortical size. Nat Genet32: 316–320. 10.1038/ng995 12355089

[15] Gimona M, Djinovic-Carugo K, Kranewitter WJ, Winder SJ (2002) Functional plasticity of CH domains. FEBS Lett 513: 98–106. 11911887 10.1016/s0014-5793(01)03240-9

[16] Korenbaum E, Rivero F (2002) Calponin homology domains at a glance. J Cell Sci115: 3543–5. 10.1242/jcs.00003 12186940

[17] Kouprina N, Pavlicek A, Collins NK, Nakano M, Noskov VN, et al (2005) The microcephaly ASPM gene is expressed in proliferating tissues and encodes for a mitotic spindle protein. Hum Mol Genet14: 2155–65. 10.1093/hmg/ddi220 15972725

[18] Higgins J, Midgley C, Bergh AM, Bell SM, Askham JM, et al (2010) Human ASPM participates in spindle organisation, spindle orientation and cytokinesis. BMC Cell Biol11: 85. 10.1186/1471-2121-11-85 PMC2988714 21044324

[19] Zhong X, Liu L, Zhao A, Pfeifer GP, Xu X (2005) The abnormal spindle-like, microcephaly-associated (ASPM) gene encodes a centrosomal protein. Cell Cycle4: 1227–9. 10.4161/cc.4.9.2029 16123590

[20] Pulvers JN, Bryk J, Fish JL, Wilsch-Bräuninger M, Arai Y, et al (2010) Mutations in mouse Aspm (abnormal spindle-like microcephaly associated) cause not only microcephaly but also major defects in the germline.Proc Natl Acad Sci U S. A107: 16595–600. 10.1073/pnas.1010494107 PMC2944708 20823249

[21] Lin SL, Qi ST, Sun SC, Wang YP, Schatten H, et al (2010) PAK1 regulates spindle microtubule organization during oocyte meiotic maturation. Front Biosci2: 1254–64. 10.2741/e187 20515799

[22] Ou XH, Li S, Xu BZ, Wang ZB, Quan S, et al (2010) p38α MAPK is a MTOC-associated protein regulating spindle assembly, spindle length and accurate chromosome segregation during mouse oocyte meiotic maturation. Cell Cycle 9: 4130–43. 20948319 10.4161/cc.9.20.13389 PMC3055197

[23] Na J, Zernicka-Goetz M (2006) Asymmetric positioning and organization of the meiotic spindle of mouse oocytes requires CDC42 function. Curr Biol16: 1249–54. 10.1016/j.cub.2006.05.023 16782018

[24] Saunders RD, Avides MC, Howard T, Gonzalez C, Glover DM (1997) The Drosophila gene abnormal spindle encodes a novel microtubule-associated protein that associates with the polar regions of the mitotic spindle. J Cell Biol137: 881–90. 10.1083/jcb.137.4.881 PMC2139842 9151690

[25] Zhu J, Qi ST, Wang YP, Wang ZB, Ouyang YC, et al (2011) Septin1 is required for spindle assembly and chromosome congression in mouse oocytes. Dev Dyn240: 2281–9. 10.1002/dvdy.22725 21932310

[26] Gaetz J, Kapoor TM (2004) Dynein/dynactin regulate metaphase spindle length by targeting depolymerizing activities to spindle poles. J Cell Biol166: 465–71. 10.1083/jcb.200404015 PMC1401226 15314063

[27] Winder SJ, Kendrick-Jones J (1995) Calcium/calmodulin-dependent regulation of the NH2-terminal F-actin binding domain of utrophin. FEBS Lett357: 125–8. 10.1016/0014-5793(94)01347-4 7805877

[28] Martin SR, Bayley PM (2002) Regulatory implications of a novel mode of interaction of calmodulin with a double IQ-motif target sequence from murine dilute myosin V. Protein. Sci11: 2909–23. 10.1110/ps.0210402 PMC2373755 12441389

[29] Wühr M, Mitchison TJ, Field CM (2008) Mitosis: new roles for myosin-X and actin at the spindle. Curr Biol18: R912–4. 10.1016/j.cub.2008.08.043 18957236

[30] do Carmo Avides M, Glover DM (1999) Abnormal spindle protein, Asp, and the integrity of mitotic centrosomal microtubule organizing centers. Science 283: 1733–5. 10073938 10.1126/science.283.5408.1733

